# Modern management of ruptured abdominal aortic aneurysm

**DOI:** 10.3389/fcvm.2023.1323465

**Published:** 2023-12-12

**Authors:** Salvatore T. Scali, David H. Stone

**Affiliations:** ^1^ Division of Vascular Surgery and Endovascular Therapy, University of Florida, Gainesville, FL, United States; ^2^ Section of Vascular Surgery, Dartmouth-Hitchcock Medical Center, Lebanon, NH, United States

**Keywords:** rupture, AAA, centralization, management, complications

## Abstract

Ruptured abdominal aortic aneurysms (rAAA) remain one of the most clinically challenging and technically complex emergencies in contemporary vascular surgery practice. Over the past 30 years, a variety of changes surrounding the treatment of rAAA have evolved including improvements in diagnosis, development of coordinated referral networks to transfer patients more efficiently to higher volume centers, deliberate de-escalation of pre-hospital resuscitation, modification of patient and procedure selection, implementation of clinical pathways, as well as enhanced awareness of certain high-impact postoperative complications. Despite these advances, current postoperative outcomes remain sobering since morbidity and mortality rates ranging from 25%-50% persist among modern published series. Some of the most impactful variation in rAAA management has been fostered by the rapid proliferation of endovascular repair (EVAR) along with service alignment at selected centers to improve timely revascularization. Indeed, clinical care pathways and emergency response networks are now increasingly utilized which has led to improved outcomes contemporaneously. Moreover, evolution in pre- and post-operative physiologic resuscitation has also contributed to observed improvements in rAAA outcomes. Due to different developments in care provision over time, the purpose of this review is to describe the modern management of rAAA, while providing historical perspectives on patient, procedure and systems-based practice elements that have evolved care delivery paradigms in this complex group of patients.

## Introduction

### Historical perspectives

Ruptured abdominal aortic aneurysm (rAAA) was first described in the 2nd century A.D. by the Greek physician Antyllus of Alexandria (1). The description included a depiction of a patient with a pulsatile abdominal mass who subsequently developed severe abdominal pain and sudden death. Although AAAs have been present throughout human history, it was not until published results by Sir Astley Cooper in the 19th century on his experiences with treating patients with this condition that this disease came to the forefront of medical knowledge (1). In fact, Cooper's writings provided Sir William Osler the necessary insights to further describe AAA patients by characterizing the clinical presentation, particularly the characteristic pulsatile abdominal mass and the propensity for rupture. In Osler's era, rare attempts at early diagnosis and surgical treatment were described, usually in the form of aortic ligation; however, mortality rates were exceedingly high (2). As improved understanding of AAAs aligned with surgical innovation, by 1923, the first successful complete ligation of an aortic aneurysm was described. Notably, the patient survived for 17 months and subsequently died from tuberculosis (2). Despite this landmark description, outcomes of aortic surgery remained dismal and a publication from the American Surgical Association in 1940 succinctly stated, “the results obtained by surgical intervention have been discouraging” (2, 3).

The modern era of rAAA management can be traced to the seminal contribution of Dr. Arthur Vorhees in 1951 (3). He performed the first documented successful open AAA repair using an in-situ aorto-aortic bypass strategy with vinyon-N graft material (e.g., material used in parachutes!). This achievement catalyzed the “golden era” of aortic surgery with significant influences imparted by Debakey and Cooley (1, 3). Although, the first case report of a successful repair of a ruptured AAA using Dacron graft was performed by Sutton in 1952, a number of technical improvements were made by Debakey, Cooley and subsequently Crawford from the 1950s to the 1970s. These included refinements in operative resuscitation, sequential clamp application, as well as selective visceral/lower extremity perfusion to mitigate the deleterious impact of ischemia-reperfusion sequelae perioperatively (1, 3).

### Epidemiology and prevalence

Although ruptured AAA is relatively uncommon compared to intact AAA, there are certain patient populations and geographic regions that have higher incidence and prevalence. It is estimated that 6%–8% of the population over age 60 have an AAA globally (4). AAA is more commonly observed in older adults with an increasing risk among men (5, 6). In fact, the prevalence for infrarenal AAAs is 4–5 fold higher for men compared to women and potentially amplified in the setting of a smoking history. Further, certain populations have higher rates of aneurysm disease especially among Northern European and North American nations which likely is influenced by genetic factors, as well as lifestyle choices (e.g., smoking) (4). The overall prevalence for AAA in multiple population based studies ranges from 3%-5% in men aged 65–70 and exceeds 10% among subjects over the age of 80. Moreover, several reported risk factors are recognized to be important contributors to AAA morbidity (6, 7).

**Figure 1:**
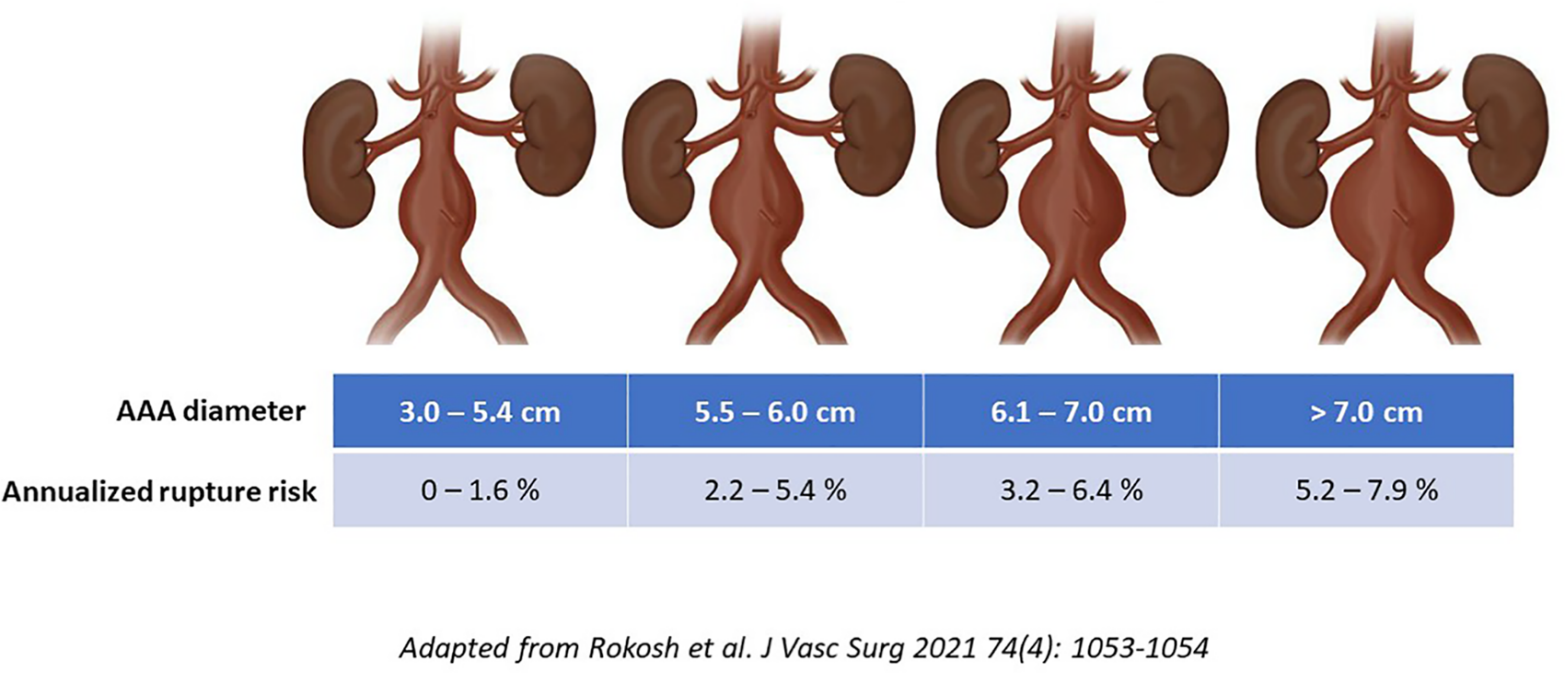
Updated AAA diameter associated annualized rupture risk estimates.

Notably, there is significant overlap among covariates that contribute both to AAA growth and rupture risk. The strongest predictors include smoking exposure (relative risk increases 3–5-fold) and family history (RR∼2-fold) (8). Additional variables such as oxygen-dependent COPD, coronary artery disease, hypertension, cerebrovascular and peripheral artery disease, as well as mixed connective tissue disorders all have associations with the natural history of AAA (9). Notwithstanding the impact of these other biologic factors that have been implicated in AAA morbidity, to date, diameter remains the single most important predictor that is used to inform perioperative rupture risk assessments. It is important to highlight that historical estimates for diameter-associated rupture risk are now recognized to have been overestimated since they were predominantly based on retrospective observations, as well as autopsy studies. This point is highlighted by the recently published North American Society for Vascular Surgery's updated guideline implementation document (10). Specifically, a 5.5 cm diameter for a man now has an endorsed annualized rupture risk of −1%–3% (which is less than the −5%–6% that was commonly espoused previously). Similarly, a 6.0 cm AAA has an approximately 3%–6% risk of rupture per year which is roughly half of what was reported in historical guidelines and series (Figure 1) (10). These revised estimates may have clinical implications surrounding recommended diameter thresholds for surgical repair.

### Risk factors and screening

Although the focus of this review is to highlight ruptured AAA management, an accounting of the relevant risk factors and further emphasis about the importance of population-based screening programs provides additional context to this discussion. The current understanding about the underlying etiology of degenerative AAA disease is characterized by a systemic process that impacts vessel wall biology leading to loss of important vascular structural proteins and strength (11). In fact, the intersection of underlying predisposing genetic factors, inflammatory mediators and proteases underscores the development of aneurysm formation. However, certain exogenous factors including tobacco use (and by extension COPD), as well as hypertension are known to further exacerbate this process.

Moreover, male sex preponderance (4–5:1 vs. female patients), increasing age, familial history (e.g., 1st degree relative), other peripheral aneurysms (e.g., iliac, femoral, popliteal), and atherosclerosis have all been recognized to be additional important risk factors for AAA development (11). Optimal medical therapies in AAA patients include longitudinal judicious blood pressure management and complete smoking/nicotine abstinence given the known direct deleterious impact this exposure has on cytoskeletal biology in the extra-cellular matrix of the aorta. Further, the use of antiplatelet agents and statin therapies are helpful to reduce long-term cardiovascular risk but there are no known pharmacologic therapies that reduce AAA growth/rupture at this time (11).

Notably, the success of smoking cessation programs at a national level have led to a decline in AAA prevalence over time. Additionally, population-based screening programs in many countries around the world have successfully reduced AAA mortality in the past 20 years (12, 13). Nationwide programs usually screen males ages 65–75 who have ever smoked and most suggest screening males aged 65–75 who have a first-degree relative who has been diagnosed with AAA. Selectively, screening can be offered to female patients who have a first-degree relative who has been diagnosed with AAA; however, screening is not otherwise indicated for women. Lastly, the Society for Vascular Surgery (SVS) and European Society for Vascular Surgery (ESVS) guidelines also suggest rescreening individuals at the 10-year time point if their original aortic diameter is between 2.5 and 3.0 cm (12, 13).

## Evolution in patient management and procedure selection

### Early recognition and diagnosis

Due to the sobering outcomes that are still reported for rAAA presentations, early diagnosis and treatment to either prevent overt rupture or rapidly triage patients with emergent symptoms remains a cornerstone of modern management. This philosophy has long been recognized to be a crucial component to the care of patients presenting non-electively with any number of acute aortic syndromes. Due to the need for timely diagnosis and treatment of the rAAA patient, emergency room providers need to have a high-index of suspicion for any patient within the 5th-9th decade of life that presents with abdominal or back pain with or without hemodynamic lability, syncope, flank/periumbilical ecchymosis and/or unexplained fall/loss of consciousness, etc. Further, any patient with a known history of AAA (either diagnosed previously or having a positive family history with first degree relatives), as well as subjects with known or suspected mixed connective tissue disease should also be considered high-risk for a rAAA presentation. This should prompt rapid acquisition of diagnostic imaging (e.g., FAST vs. CT angiography with CT being the gold standard) to rule out the possibility of an aortic emergency.

**Figure 2:**
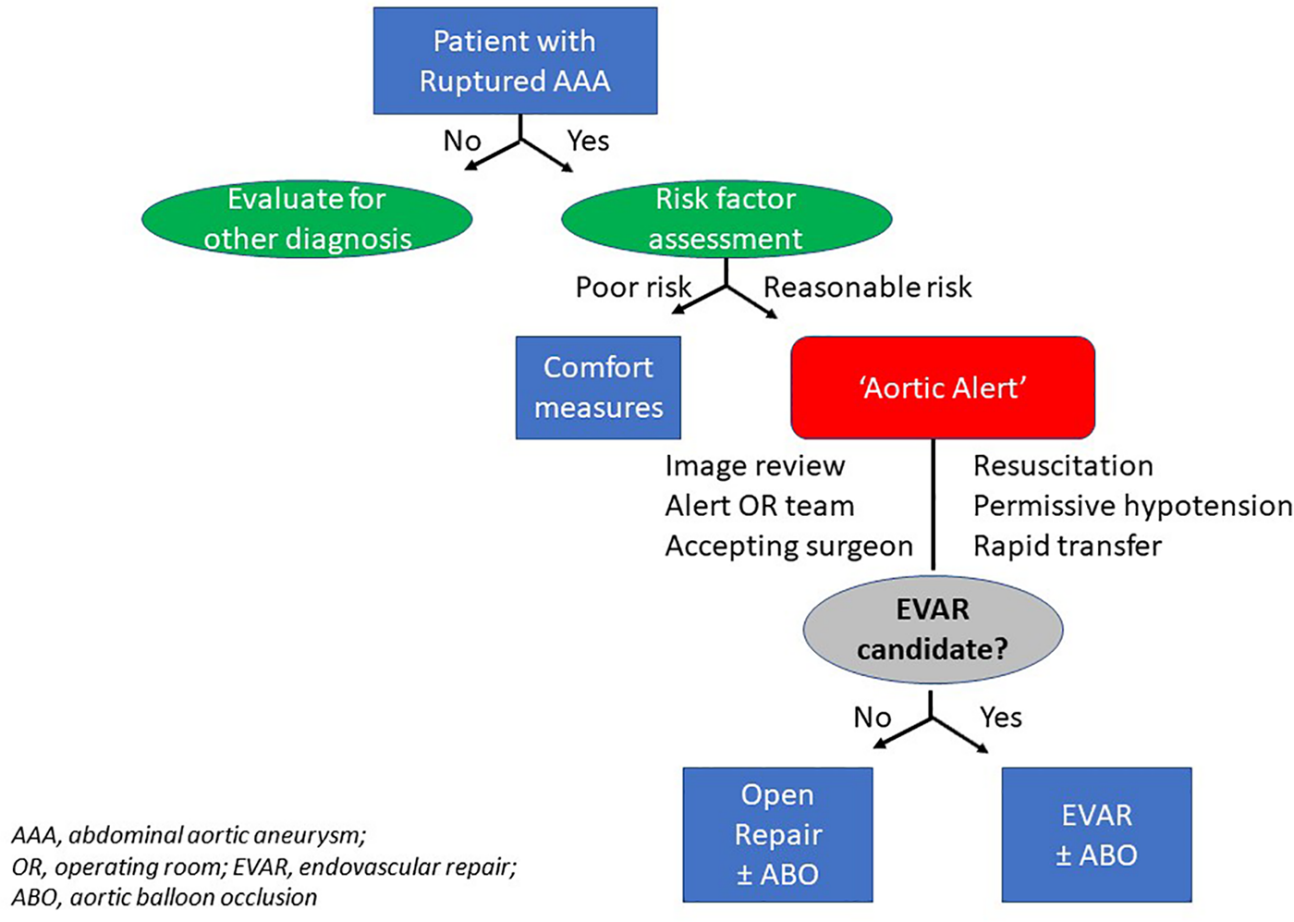
Ruptured AAA management algorithm.

In an effort to coordinate care and expediently transfer and/or treat rAAA, there are several descriptions of large healthcare networks that have successfully implemented ‘hub and spoke’ models to centralize care of these patients (14, 15). The concept can be generally described as follows: once a smaller hospital or satellite emergency room has identified a patient to have a rAAA, there are clear process of care algorithms (Figure 2) that are enacted similar to treatment of patients experiencing acute coronary syndromes or stroke. In fact, this strategy has been successfully employed in different regions of the United States, as well as multiple European and Australasian nations (16, 17).

The value of these protocol driven care pathways for rAAA patients is that it provides non-vascular health care practitioners with the necessary information to appropriately diagnose, notify, support and rapidly transfer patients to larger more complex tertiary referral “centers of excellence” for definitive management. Secular regionalization of rAAA care has occurred in North America; however, this unfortunately has not been uniform in its application (18). Unlike Canadian, most European, and some Australasian nations that have single-payor systems that can mandate regionalization, a predominantly fee-for-service health ecosystem and an overriding doctrine to preserve both patient and physician autonomy has been associated with the unintended consequence of slowing centralization within the United States (19). This distinction is somewhat ironic since the original description of a center volume association with postoperative outcome relationship after major cavitary and extirpative surgery was actually described by Luft and colleagues in 1979 from their analysis of patients treated in California (20). Additional barriers to regionalization of rAAA management in the U.S. have included complex geographic, financial, and patient-related factors which has led to growing calls from professional societies to incentivize centralization models both in the U.S and internationally (12, 18, 21).

Despite this uneven adoption of rAAA care regionalization, the upstate New York experience highlights the successful implementation of a coordinated care network among 12 different hospitals (22). For example, over a 13-year period, a multi-disciplinary protocol was derived through collaboration among surgeons, emergency room physicians, anesthesiologists, nurses and radiology technicians. Virtual platforms for image sharing, as well as resuscitative physiologic parameters were prospectively prescribed. Interestingly, the likelihood for exposure to a specific treatment strategy (e.g., open vs. EVAR) was vastly different depending on where the operation was performed (community hospital-94% open vs. university ‘hub’ hospital-38% open). More importantly, transferred patients, had significantly improved mortality outcomes compared to their community-treated rAAA counterparts (e.g., 30-day mortality: university open repair, 27% vs. community open repair, 46%) (22). Although the difference among EVAR treated patients was less evident, there was still a strong trend toward improved morbidity, length of stay, and reintervention outcomes for the ‘hub’ hospital endovascular repairs compared to the referral network institutions. Interestingly, the results of this regional success in New York state has been demonstrated at the national level in several European countries further validating the success of this modern care model for rAAA (17).

### Pre-hospital and emergency department management

Current strategies that are now routinely employed for the pre-hospital and emergency department management of a rAAA are informed by lessons extrapolated from military and civilian trauma experiences. Modernization of trauma care delivery systems in the late 20th century included advances in the assessment and management of both blunt and penetrating trauma victims (23). State of the art principles exported from trauma networks into rAAA care algorithms now include empiric application of supplemental oxygen, large bore intravenous access (e.g., bilateral antecubital fossa, 14–16 gauge), permissive hypotension (e.g., SBP 70–90 mmHg), restrictive isotonic crystalloid infusion, and (if available), occasionally external anti-shock garments (12). Optimally, some permutation of a health information portability and accountability act (HIPAA)-compliant “aortic alert system” can be rapidly activated upon recognition and during the process of hospital transfer (4, 12, 22). This notification alerts the on-call vascular surgeon, anesthesiologist, operating room team, blood bank personnel and critical care teams. Information including the patient name, age, intravenous access, hemodynamic status (including use of vasopressors and airway status), level of consciousness, pre-hospital cardiopulmonary resuscitation history, code status and estimated time of arrival are some of the fundamental components that are usually needed to transfer the patient to the appropriate level of care (4, 17, 22).

### Evolution in patient selection

Proper patient selection is crucial to all surgical endeavors and rAAA is no exception. Patients frequently present with advanced age and may have associated hemodynamic instability and profound metabolic derangements. Therefore, it is incumbent on both the referring and accepting physicians to have a clear understanding of not only the current physiologic status of the patient but also (if possible) some insight about the relevant medical history and patient/family goals of care. Optimally, emergency room providers should address goals of care with a patient and their family (assuming hemodynamic stability) to obviate unnecessary transfer, cost, and waste of important resources. There are anecdotal descriptions of modern health system integration with technology being leveraged in these situations (4). The concept being that this would facilitate real-time accepting surgeon-to-patient phone conversation (e.g., “face time” or telehealth visit) as a way for the referral center to interact with the subject before transfer to ensure an attempt at rAAA repair along with all of the postoperative risk and potential sequelae are truly something they want to pursue. Although this sounds ideal, in reality, this rarely occurs since vascular surgeons managing rAAA patients are usually confronted with a complex multi-dimensional problem that requires rapid decision-making to arrive at an optimal treatment plan that often has to be enacted before the patient even arrives (e.g., operating room team preparing instrumentation, anesthesia-surgeon communication, ICU bed creation, etc.).

Not surprisingly, a number of preoperative risk stratification tools (e.g., Glasgow Aneurysm Score, Hardman Index, VQI rAAA score, etc.) have been published to facilitate the clinical decision making between surgeons and patients with rAAA and quantify surgical risk in this tenuous cohort (7, 15, 24–26). Although none have been shown to be “optimal”, consideration of age, anatomic complexity (e.g., need for supra-mesenteric cross-clamp), pre-hospital hemodynamic instability and/or loss of consciousness have been consistently reported to be high-risk variables that reliably predict poor outcomes after rAAA repair. For example, Robinson and colleagues identified that rAAA patients with all 4 of these risk factors had a 90%–100% mortality outcome with attempted repair (27). Similarly, the University of Washington rAAA risk score has been shown to be associated with >80% mortality if patients have age >76, SBP <70 mmHg at any time preoperatively, serum creatinine >2.0 mg/dl and serum pH <7.2 (28) (Table 1). This identifies a subset of patients that should probably be considered futile and deemed inoperable. However, a majority of patients will have some constellation of different risk factors so risk-assessment tools alone should not supplant judgment of the operating surgeon. Moreover, despite numerous publications, the widespread adoption of these risk predictor adjuncts remains scant in daily practice.

**Table 1:** Preoperative risk prediction scores for ruptured AAA.

Risk score name	Variables in model	Mortality risk with all factors
Hardman index	Age >76, Hgb<9 g/dl, Cr>190 umol/L, LOC, cardiac ischemia	100%
Glasgow aneurysm score (updated)	Age, cardiac/stroke, shock, renal disease, open repair	>90%
Vancouver score	Age, LOC, cardiac arrest	%
Edinburgh rupture score	GCS<15, SBP<90, HB<5.6 mmol/L	>80%
VSGNE score	Age > 76, cardiac arrest, LOC, supraceliac clamp	>85%
Ruptured AAA score	Age, Creatinine, SBP	65%
Dutch aneurysm score	Age, lowest SBP, CPR, Hgb level	83%
Weigarten score	Hypotension, cardiac arrest, LOC, intubation	–
Artificial neuronal network	Age ≥ 70, LOC, cardiac arrest, shock	89%
Harborview risk score	Age > 76, Cr >2.0, SBP <70, pH <7.2	100%

### Imaging

**Figure 3:**
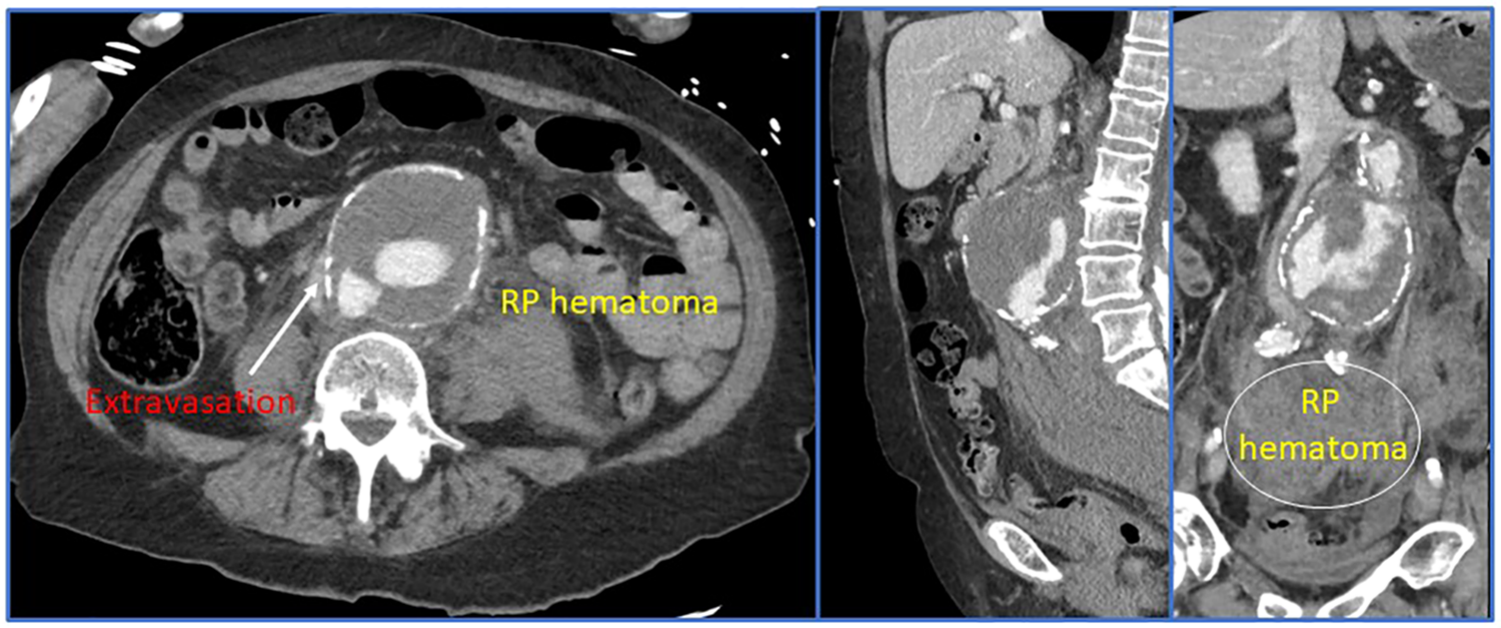
Preoperative and intraoperative imaging for a ruptured abdominal aortic aneurysm.

Some of the most important advancements in the modern management of rAAA can be linked to improvements and innovation in preoperative and intraoperative imaging. The gold-standard for imaging is thin-sliced (<1.0 mm) computed tomographic angiography with reformatted images (Figure 3). Most modern healthcare systems across different nations now have access to these scanners and the authors consider it mandatory that patients have a CTA before arrival to the operating room. This remains true even in the hemodynamically unstable patient—since without appropriate imaging, the aneurysm extent, associated morphology, and assessment of the paravisceral aorta would be unknown. Blindly operating on a rAAA that was diagnosed using point-of-care ultrasound is not recommended especially since delineation of relevant anatomic characteristics informs the operating surgeon about the optimal treatment modality and surgical approach (e.g., open vs. endovascular repair; if open, retroperitoneal vs. transperitoneal, etc.). Rapid imaging acquisition times now permit obtaining this vital anatomic information with minimal delay toward operative repair. However, we concede that there are some centers (29) who will allow patients with rAAA to proceed to the operating room without a CT scan and may achieve good results. Irrespective of what imaging bias is employed, an efficient care system is required to ensure timely delivery of the patient to the operating theater.

Due to the increasing adoption of endovascular techniques to manage the entire spectrum of acute aortic syndromes, many high-volume centers routinely employ three dimensional modeling for rAAA patients using a number of different software platforms (e.g., Siemens Syngo.via®, Osirix^™^, 3Mensio Vascular®, or TeraRecon, Inc., etc.). Contemporary tertiary aortic referral centers commonly have their own cloud sharing services that allow referring hospitals to rapidly upload images while patients are being transferred. The accepting surgeon can then take these images, generate a 3D-model and determine anatomic eligibility for attempted endovascular repair. Moreover, this designation helps the operating room team so it can set-up the appropriate equipment and instrumentation.

To further highlight advancements in imaging, modern care of the rAAA patient now increasingly utilizes fixed imaging suites that have on-table, real-time software to allow for hybridized CT-fusion imaging, as well as intravascular ultrasound that are useful adjunctive techniques that can often facilitate endovascular repair. The caveat being that the patient has enough hemodynamic stability and that the processes of care at the treating center efficiently employ these novel imaging tools without delaying definitive repair.

## Perioperative physiological management

### Hemodynamic support

Initial resuscitation in the operating room immediately before repair should generally follow the philosophy highlighted previously in the preoperative phase. Therefore, permissive hypovolemia with controlled hypotension (e.g., target SBP <90 mmHg) with balanced transfusion (e.g., whole blood or “1:1:1” infusion of PRBC/plasma/platelets), active warming and minimization of aggressive crystalloid administration are guiding principles. Recent evidence has suggested that higher ratio of plasma to packed red-cell transfusion may be associated with improved survival after operative management of rAAA but the quality of evidence has been assessed to be low with significant reporting bias. The rationale for minimizing isotonic crystalloid provision perioperatively is extrapolated from observations regarding postoperative resuscitation pattern association among trauma patients. Specifically, reliance on whole blood or 1:1:1 blood product support instead of crystalloid has led to preventing coagulopathy, multi-system organ failure and death. Moreover, preoperative dilutional coagulopathy and bolused infusion of crystalloid is thought to either precipitate or exacerbate bleeding with rAAA and should therefore be avoided.

**Figure 4:**
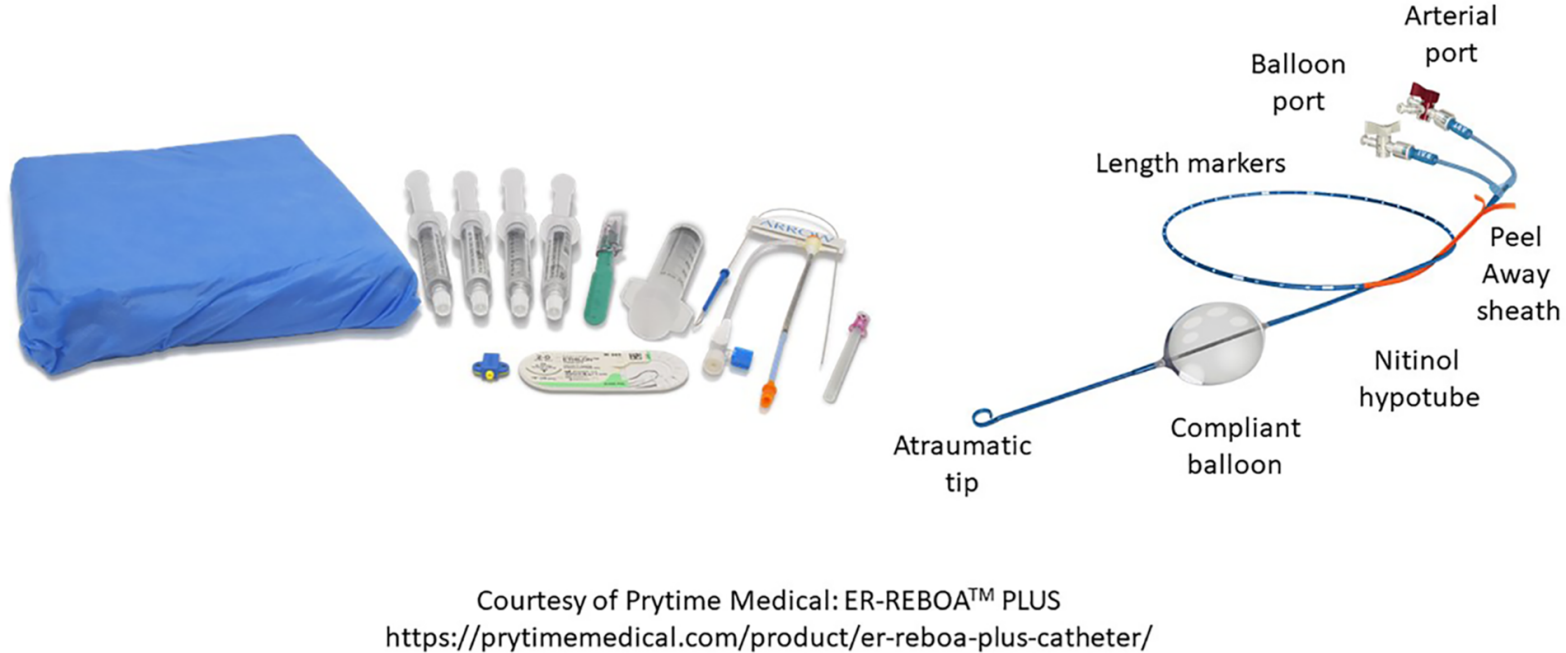
Components of a REBOA kit.

Upon arrival to the operating room, the attending surgeon and anesthesiologist need to have a clear plan about the timing of anesthetic induction since hemodynamic collapse can occur when general anesthesia is instituted. Accordingly, it is the author's recommendation that endotracheal intubation not occur until the patient is adequately positioned with appropriate lines placed and the operative field is established (e.g., “prep and drape” the awake patient). Surgeon preference dictates use of resuscitative endovascular balloon occlusion (e.g., ‘aortic balloon occlusion or REBOA; Figure 4) which can facilitate either open or endovascular repair (*see aortic balloon occlusion section below*). Moreover, this can be placed under local anesthesia prior to induction of general anesthesia should hemodynamic instability ensue. Subsequently, as the operation progresses, communication between the anesthesiologist and vascular surgeon further informs needs to escalate resuscitation depending on the hemodynamic, rheologic, thermodynamic and coagulopathic state of the patient.

### Coagulopathy and laboratory parameters

Use of thromboelastography or pulse-pressure variation in mechanically ventilated patients is now commonly employed to guide intraoperative and postoperative resuscitation in the critically ill patient. A preoperative exposure history to direct oral anticoagulants, antiplatelet agents, as well as warfarin is not uncommon due to the frequency of associated cardiovascular risk factors among rAAA patients. Accordingly, complete blood count, coagulation profiles, and comprehensive metabolic panels including hepatic function are standard assessments upon arrival to the accepting facility. Additionally, arterial blood gas analysis with lactate and base deficit estimates are used to further guide goal-directed therapy throughout the perioperative period. Importantly, the most critical “lab test” to obtain before arrival to the operating room is a type and cross-match since 30%–50% of rAAA patients will receive transfusions.

### Cardiac evaluation and comorbidity assessment

Due to the non-elective presentation, it is common for patients to have no preoperative information about their pre-existing baseline cardiopulmonary function. It is assumed that all AAA patients harbor some level of coronary atherosclerosis and due to the high prevalence of historical or current tobacco exposure, significant COPD may impact perioperative management. These conditions along with possible acute and/or chronic renal disease have significant implications on anesthetic choice, dosing and metabolism. Because of the unique metabolic and physiologic signatures that rAAA patients can harbor, centers that perform high-volume aortic surgery often have dedicated cardiovascular anesthesia trained physicians involved in these cases. Moreover, intraoperative transesophageal echocardiography can provide real-time feedback to both the anesthesia and surgical team to help guide resuscitation. This is especially true after release of an aortic cross-clamp (or aortic balloon occlusion; ABO) once a(n) (endo)graft is placed since ischemia-reperfusion phenomenon can cause profound hemodynamic lability which requires rapid management with coordination between teams. Direct “closed loop” communication between the anesthesiologist and surgeon can provide insights about any observed coagulopathic bleeding, on-going surgical bleeding, as well as physiologic responses to attempted removal of the aortic cross-clamp.

### Aortic balloon occlusion

**Figure 5:**
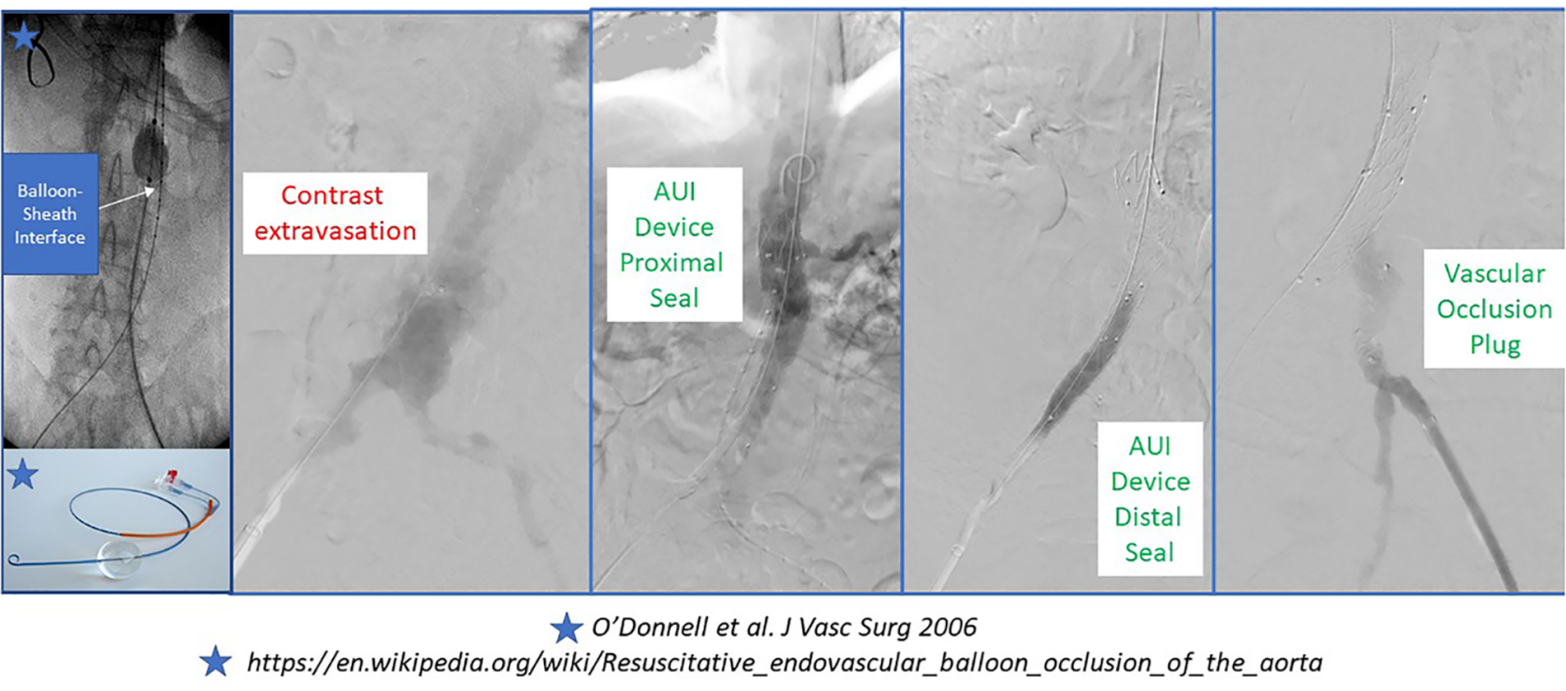
Intraoperative imaging during ruptured endovascular abdominal aortic aneurysm repair.

Many modern series of rAAA repair now highlight the feasibility, safety and utility of selective endovascular ABO. The original descriptions pre-dated the REBOA experience that is now popularized in the trauma literature. Many authors describe the use of percutaneous transfemoral access in the awake patient to position a long-sheath with a stiff wire (e.g., Amplatz or Lunderquist) and a compliant molding balloon above the renal or mesenteric arteries (depending on aneurysm extent and status of the paravisceral aorta) using fluoroscopic guidance. The molding balloon is positioned appropriately and then a repair can follow with communication between the surgeon and anesthesiologist about whether the case will be completed under local anesthesia (e.g., EVAR) or if conversion to general anesthesia for open repair is needed (30). In the latter scenario, aortic balloon occlusion is only applied if the patient has rapid hemodynamic deterioration with anesthetic induction (Figure 5) (31).

Several different depictions for ABO during rAAA have been reported including ‘double-balloon’ techniques during EVAR. Upper extremity and lower extremity percutaneous or open arterial access have been reported with transfemoral access being most prevalent. Historically, a 12–14Fr 45 cm sheath was employed; however, modern integrated REBOA systems are now as low profile as 7Fr (31).

## Open surgical treatment

### Indications for open repair

rAAA patients that are deemed to be unsuitable for EVAR (see next section) should undergo open surgical repair (Table 2). Preoperative planning requires consideration of a number of variables that account for important hemodynamic and anatomic features of the presentation. The operating surgeon needs to decide on the surgical approach (e.g., midline laparotomy vs. retroperitoneal access), identify the proximal and distal cross-clamp locations, and account for the presence of relevant anatomic factors (e.g., calcification, prior colectomy, presence of hernia/stoma, retro or circum-aortic renal vein, concurrent iliac aneurysm, aorto-caval fistula, duplicated or left-sided inferior vena cava, horseshoe kidney, renal/mesenteric vessel patency, etc.). Some centers have favored one approach over another (e.g., retroperitoneal > transperitoneal) and it is the author's contention that retroperitoneal access provides the greatest flexibility with proximal aortic cross-clamp application. However, some surgeons are less familiar with this technique and prefer a transperitoneal incision, which is reasonable as long as renal-mesenteric revascularization is not anticipated. Despite these differences, it should be highlighted that retroperitoneal access mandates positioning the patient in such a manner that it may impair performance of CPR, as well as aorto-femoral (e.g., Right femoral access) reconstruction.

**Table 2:** Anatomic considerations for ruptured abdominal aortic aneurysm repair perioperative planning.

Open repair	Endovascular repair	Additional considerations independent of proposed strategy
Aneurysm extent (e.g., TAAA/suprarenal)	Aneurysm extent (e.g., infrarenal/juxtarenal)	Mesenteric/renal disease
Supraceliac aorta (cross-clamp suitability)	Parallel/healthy proximal LZ	Previous colectomy/collateral
Peri-renal/visceral calcification	Infrarenal neck ≥10 mm	IMA and IIA patency
Concurrent aorto-iliac occlusive disease	*Β*-angle ≤75 degrees (optimal 60)	Previous EVAR or Open repair
Location of hematoma (e.g., free rupture, contained within retroperitoneum)	Neck diameter ≤32 mm	
Retro-aortic or circum-aortic left renal vein	Terminal aortic diameter ≥10 mm	
Horseshoe kidney, inflammatory AAA	Iliac diameter ≥5 mm	
Left sided IVC	Iliac tortuosity/calcification	
Concurrent iliac aneurysm	Iliac/femoral access (scar/occlusive disease/calcium)	

### Procedural steps

After anesthetic induction, which may or may not be bridged physiologically using ABO, the surgical team performs either a midline laparotomy or a left (thoraco) retroperitoneal incision. Early identification and control of the supraceliac aorta is achieved; however, a cross-clamp is only applied if the patient is unstable. Next, the pararenal aortic dissection proceeds and if a suitable clamp location at or above the renal arteries is identified, the clamp is transferred to this position. Care is taken to minimize dissection of the iliac vessels to avoid injury to the iliac veins. Depending on the coagulation profile of the patient and overall stability, intravenous heparin can be given prior to placing the distal cross-clamp followed by application of the proximal cross-clamp (if hemodynamically stable; however, if unstable the proximal clamp is applied first). The aneurysm is opened longitudinally and an appropriately sized Dacron graft, which is informed by preoperative assessment of the CTA and intraoperative inspection of the aortic diameters, is selected. An in-situ aorto-aortic or aorto-iliac (or femoral) reconstruction is then completed and decisions about internal iliac and/or IMA revascularization are considered to reduce risk of pelvic/colonic ischemia but depend on the physiologic state of the patient.

Next, heparin anticoagulation is reversed with protamine and care is taken to assure hemostasis. Finally, it is strongly recommended to use “damage control” principles and a planned 2nd look operation after open repair of rAAA so deliberate placement of hemostatic laparotomy sponges and a temporary vacuum-assisted closure dressing is advised. This strategy helps reduce risk of abdominal compartment syndrome, mitigate consumptive coagulopathy that can be precipitated by hematoma formation and affords the opportunity to inspect the bowel viability after a planned period of resuscitation.

## Endovascular treatment

### Indications for endovascular repair

**Figure 6:**
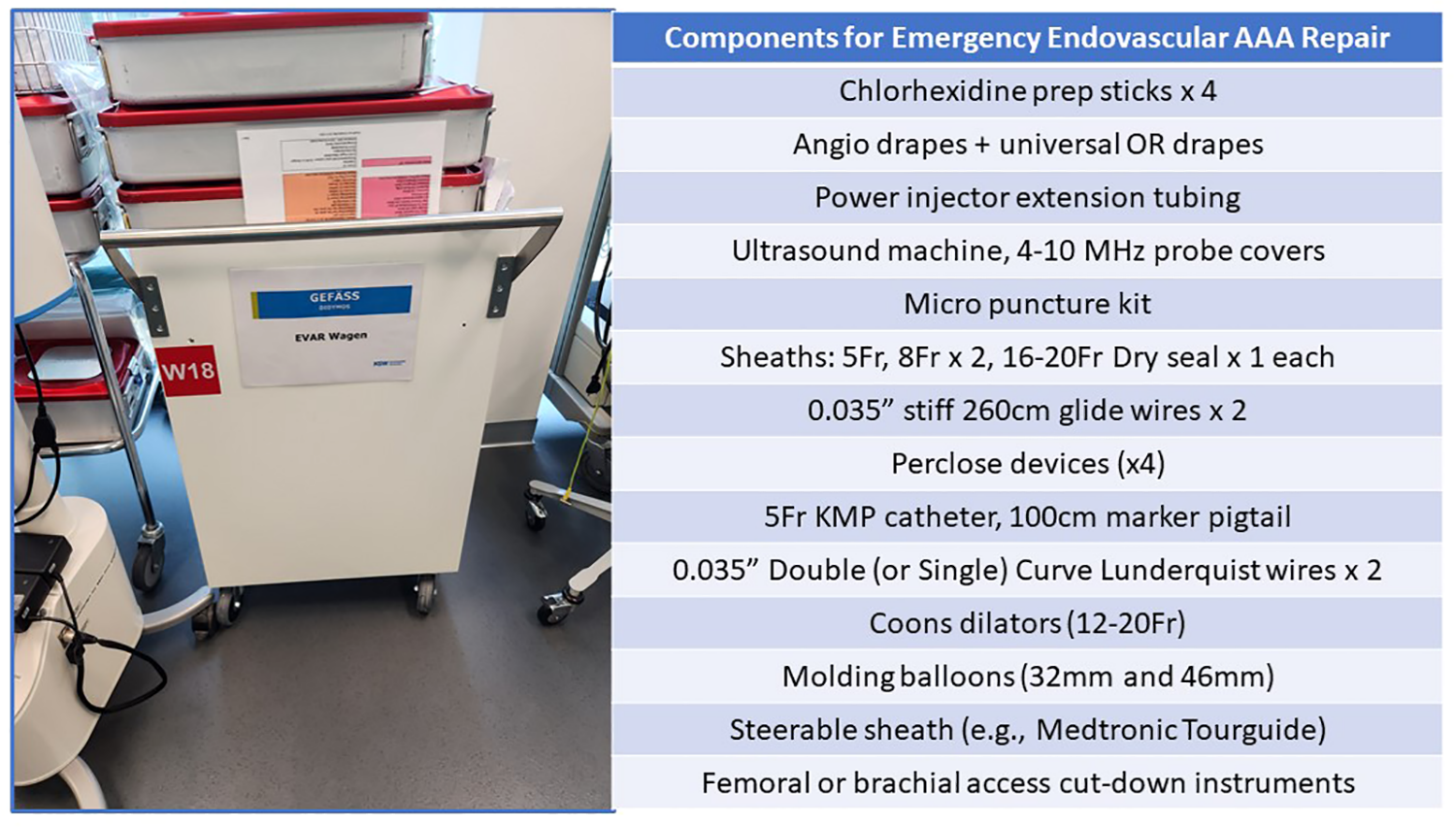
Endovascular ruptured AAA repair kit.

Many centers now employ EVAR as their preferred treatment strategy for rAAA patients (15, 32, 33). Eligibility for endoluminal management usually can be assessed using the preoperative CT imaging (Figures 3, 5). Various series have identified that when using “extended anatomic criteria”, approximately 60%–70% of rAAA patients qualify for EVAR. Inflection points to consider in the decision-algorithm about whether or not to employ a stent-graft are influenced by the aortic neck diameter (e.g., ≤32 mm), infrarenal neck length (e.g., ≥10 mm), as well as *β*-neck angulation (≤75^0^) (4, 32). Importantly, oversizing principles generally are on the upper end of the 20%-30% spectrum since many patients have hypovolemia and underfilling of their aorta on presentation which should be considered in proximal endograft sizing. Further, in select centers, use of physician-modified EVAR or parallel/chimney stenting techniques has increased the spectrum of patients that are now deemed eligible for emergent EVAR. Given the complexity involved in these cases, having the necessary stent-graft inventory along with the ancillary wires, catheters, sheaths and operative team experience to support use of these technologies is paramount to success. Accordingly, to mitigate case preparation time, many centers now have “rupture kits” (Figure 6) which have all the fundamental endovascular (and open) surgical tools pre-selected and aggregated so that they are ready for rapid deployment.

### Procedural steps

The surgeons’ decision to use a particular stent-graft is most often determined by the patient's anatomy though certain scenarios can influence use of bifurcated vs. aorto-uni-iliac (AUI) stent grafts. For example, if a bifurcated endograft is employed in an unstable patient, on-going bleeding is occurring while attempts are made with gate cannulation. Therefore, iliac artery tortuosity and stenotic disease needs to be considered before the implant choice is made. If attempts at gate cannulation take too long and the patient has on-going hemodynamic instability, a decision to convert to an AUI configuration needs to occur quickly which necessitates implanting either a converter device or an aortic cuff across the flow divider. Ultimately, contralateral iliac occlusion is still required followed by a femoral-femoral bypass. In the author's experience, if attempting a bifurcated device, intentionally positioning the contralateral gait into a cross-limb configuration and positioning a steerable sheath immediately below the gait can facilitate cannulation. Alternatively, some centers use AUI devices almost exclusively when performing EVAR of rAAA because of these concerns.

A variety of adjunctive techniques may be necessary to assure adequate seal and fixation of an endograft when managing rAAA. Pre-implantation intraoperative angiography to mark the relevant anatomy is routine (with or without ABO above the renal-mesenteric vessels); however, selective utilization of intravascular ultrasound can add useful information to verify device sizing, positioning, gait cannulation and endograft effacement. Since many scenarios may be characterized by having “suboptimal” anatomy especially involving the proximal landing zone, use of aortic cuff extension or endo-anchors may be necessary to achieve a satisfactory technical result. On completion imaging, type 1 or 3 endoleak must be treated with decisions informed by anatomy, surgeon-skill set, implant inventory and physiologic status of the patient. However, type II endoleaks present a unique dilemma for ruptured EVAR cases. There is limited data or consensus on how best to manage this issue. The authors only perform decompressive laparotomy for rupture EVAR cases in patients with abdominal compartment syndrome so most type II endoleaks are not treated at the index operation. Notably, if patients have “free” intraperitoneal rupture, it has been the author's experience that type II endoleak can be more problematic which may lead to selective sac exploration and over sewing after implant of the endograft. Finally, decisions about decompressive laparotomy need to be made since retroperitoneal bleeding that occurred intraprocedurally or from lumbar arteries can lead to mass effects that precipitate abdominal compartment syndrome (ACS). Prolonged supraceliac ABO (e.g., >30 min) and massive intraoperative transfusion (e.g., >10 PRBC units) are highly predictive of ACS and generally should lead to empiric laparotomy (33). Alternatively, if a selective approach is employed, serial postoperative bladder (e.g., >20 mmHg) and peak airway pressure (e.g., >45 cmH_2_0) assessments along with vigilant clinical judgment are needed (4, 34).

## Postoperative care & outcomes

### ICU management

During the early postoperative period, rAAA patients often require significant fluid resuscitation and rewarming. This is true for both EVAR and Open AAA patients. Patients generally leave the operating room intubated and may or may not need vasopressor infusions to augment on-going blood product and crystalloid transfusion. Close monitoring of thermoregulation, arterial blood gas, base deficit, lactate, pulse pressure variability, central venous pressure, urine output, heart rate, mean arterial pressure, pulmonary physiology, as well as renal, hepatic and coagulation functional assessments is standard. Goal-directed therapies and protocols are commonly employed which reacts to the patient's dynamic physiologic state.

If the patient has a planned “2nd look” operation, this typically occurs within 1–3 days after the index rAAA repair. Optimally, the patient returns once they have achieved better physiological resuscitation at which time any retained laparotomy sponges are removed. The decision to close the fascia at this stage is usually based upon impressions of the abdominal wall compliance and status of the patient's respiratory mechanics. Once the incision is closed, postoperative convalescence is generally informed by usual ICU milestones for neurologic, cardiac, pulmonary, gastrointestinal, renal, and hematologic parameters (4, 34).

### Complication surveillance after rAAA repair

Independent of which strategy is employed to manage rAAA, patients commonly experience postoperative complications (e.g., >50% incidence). Although it is not an exaggeration to say virtually any and all complications can occur to a patient after a rAAA repair, modern management has identified certain high-impact events that warrant either dedicated monitoring, elevated index of suspicion or both. For example, aside from the aforementioned ACS, colonic ischemia and acute kidney injury are notable morbidities to review.

Colonic ischemia occurs at an incidence of <1% after elective EVAR procedures and rates of 1%–3% are commonly reported for intact open AAA repair (12). By comparison, incidence of colonic ischemia after rAAA can exceed 20% in some series depending on how it is defined and what surveillance mechanism is implemented (4). Also, it is important to highlight that open repair appears to have a 2–3 fold higher risk of colonic ischemia compared to EVAR for rAAA presentations (16). These observations have led some centers to use routine flexible sigmoidoscopy for postoperative detection. Irrespectively, once the diagnosis is considered, aggressive management is warranted since mortality risk increases >3–5 fold (16).

Next, the incidence of in-hospital acute kidney injury from acute tubular necrosis after rAAA repair exceeds 30%-50% and is due to a number of causes including hemodynamic lability, cardiac dysfunction, intravenous dye/antibiotic exposure, use of ABO and/or suprarenal cross-clamp, as well as pre-existing renal disease, among others (35). One must also consider a period of potential renal hypoperfusion prior to presentation which may augment the injury. Not surprisingly, this portends a negative short and long-term prognosis for the rAAA patient. ICU monitoring and proactive interventions to support cardiac and renal function are now increasingly built into clinical pathways for recovering rAAA subjects. Moreover, intraoperative maneuvers to mitigate renal ischemia time are crucial but are balanced by the technical and physiologic demands of the rAAA procedure. Unfortunately, rates of temporary or permanent dialysis can range from 5%-15% in some populations which significantly impacts fluid balance and cardiopulmonary performance during the post-operative period (35). Early use of continuous veno-venous hemofiltration, as well as urine and serum biomarkers (e.g., SLP1, IGFBP7) to detect pre-clinical changes in renal function are increasingly reported (36).

There are a number of other important complications including (but not limited too) bleeding, arrythmia, myocardial infarction, congestive heart failure, and respiratory failure that may occur after rAAA management. Although an exhaustive review of each is beyond the scope of this review, the following section provides a contemporary perspective on their frequency and associated impact on outcomes.

### Mortality outcomes

The results of rAAA management have steadily improved over the past 30–40 years but seem to have plateaued somewhat in the most recent decade. The underlying mechanism to explain this is multi-factorial given the changes in regionalization, resuscitation strategy, institutional expertise, EVAR adoption, and recognition of certain complications. The unfortunate reality is that up to 2/3rd of patients with a rAAA never make it to a hospital and as many as 20% are not able to be transferred to a center that can perform a definitive repair (4, 34, 37). This sobering reality combined with the high incidence of postoperative complications and mortality is the reason rAAA has an 80% mortality rate associated with the diagnosis (4, 34). However, this is often misinterpreted when published series of rAAA outcomes are discussed since 50%–75% 30-day survival among operated patients is often cited. The important distinction being that the ascertainment, referral and selection bias for subjects to survive long enough to undergo definitive repair is rarely accounted for in those results.

Once a patient reaches a center and is deemed operable, the following description provides a perspective on contemporary results of rAAA repair outcomes. Most commonly, series provide comparisons of EVAR and Open repair mortality outcomes. 30-day mortality estimates range from 15%-50% with many institutional series documenting an advantage with EVAR. Indeed, there has been intense investigation into the potential mortality advantages of EVAR vs. open repair for rAAA but to date, the evidence is probably best characterized as being indeterminate. For example, a large retrospective claims cohort study of 3,164 rAAA patients treated in the United States from 2009 to 2015 reported that in-hospital mortality was significantly lower for EVAR when compared with open repair (23.8% vs. 36.3%) (38). A similar analysis of the Vascular Quality Initiative also appeared to identify the survival advantage (23% vs. 35% for EVAR vs. open repair) (39). However, to date, these results have not been consistently validated in higher levels of evidence including different large randomized multi-center trials (Table 3).

**Table 3:** Outcomes of ruptured abdominal aortic aneurysm repair in selected modern reported series.

Study	Patients	Open Repair 30-day mortality	EVAR 30-day mortality	Comments
Veith et al. 2009	1,037	36.3%	21.2%	Retrospective, multi-center review
Giles et al. 2009	28,429	41%	33%	National Inpatient Sample, U.S. cohort, inpatient mortality
Starnes et al. (32)	187	54.2%	18.5%	Single center, retro & prospective
Nedeau et al. 2012	74	49.0%	15.7%	Single center, retrospective
Reimerink et al. (40) (AJAX)	116	25%	24%	Small RCT, stable patients, restrictive anatomic & physiologic inclusion
Karthikesalingam et al. (7)	11,799* vs. 23,838	45%–65%	35%–50%	England* (Hospital Episode Statistics) vs. U.S. (National Inpatient Sample) comparison
Edwards et al. 2014	1,099	47.7%	33.8%	Medicare population, propensity score match comparison
Mohan et al. 2014	42,126	39.1%	25.9%	National Inpatient Sample, Updated U.S. Cohort, in-hospital mortality
Desgranges et al. 2014 (ECAR)	107	19%	22%	Small RCT, stable patients, restrictive anatomic & physiologic inclusion
Powell et al. 2014 (IMPROVE)	613	37.4%	35.4%	Pragmatic, large RCT, intention-to-treat analysis, no short term but 3-yr EVAR survival advantage
Rango et al. 2016	55	63%	42%	Single center, retrospective
Tan et al. 2017	1,048	47%	33%	ACS-NSQIP; octogenarians
Gupta et al. (38)	3,164	36.3%	23.6%	Retrospective, claims analysis
Roosendaal et al. 2020	7,526	–	–	Systematic Review & Meta-analysis: OR 0.5, 95% CI .38–.67, EVAR>OAR
Greenleaf et al. 2020	2,895	–	–	Vascular Quality Initiative Registry: High-volume Center 33% lower mortality with open repair; no volume-effect with rEVAR
Jones et al. 2022	376	29.9%	27.7%	Single center, retrospective
Pomy et al. 2022	7,547	40%–41%	–	NSQIP, worsening open repair outcomes due to bleeding/MACE

* Highlights the 11,799 patients which correspond to the English healthcare system.

The Acute Aneurysm Trial examined outcomes among 116 patients but excluded subjects with hemodynamic instability (40). Similarly, the Endovasculaire ou Chirurgie dans les Anévrysmes aorto-iliaques Rompus trial (ECAR) enrolled 107 patients with hemodynamic stability (41). Both trials had low 30-day mortality outcomes overall (21% and 25%, respectively) with no clear benefit of either EVAR or open repair. In contrast, the Immediate Management of Patients with Rupture: Open Versus Endovascular Repair (IMPROVE) trial expanded enrollment criteria to include any patient with a diagnosis of rAAA (42). Interestingly, among the 613 study patients, only 174 met the anatomic inclusion criteria to undergo EVAR even though 316 were initially randomized to the endovascular arm. The overall short term mortality outcomes were similar (EVAR, 35.4% vs. Open, 37.4%); however, the trial design and strict anatomic criteria for ultimate treatment for EVAR with the intention-to-treat *a priori* analysis has been significantly critiqued since it did not seemingly reflect real-world decision-making. Moreover, the 3-year mortality outcomes actually favored the endovascular cohort (48% vs. 56%, open).

When examining long-term outcomes of rAAA repair, historical analysis of the Swedvasc registry provides an excellent benchmark. Specifically, among more than 4,000 rAAA repairs that were performed from 1987 to 2005, long-term survival remained largely unchanged over time (e.g., 5-year crude survival: 41.7%, 95% CI 39.6–43.7%) (43). Interestingly, these results were not associated with either patient sex or specific age groups. In addition to survival, patient quality of life (QOL) has often been highlighted to be an important parameter to judge clinical success of rAAA repair and Yildrim and colleagues determined that 5-year outcomes are equivalent to age-matched controls (44). Lastly, when comparing differences in long-term outcomes for rAAA patients receiving either open repair or EVAR, due to the inherent anatomic and physiologic factors that influence decisions about treatment options, it is difficult to draw definitive conclusions.

Further, some authors contend that long-term survival is comparable between open and endovascular patients; however, higher reintervention rates and risk of conversion are important features differentiating these strategies (45, 46). Notwithstanding these confounding aspects of management decisions surrounding rAAA care provision, the mid-term results of the IMPROVE trial did document improved survival and QOL for patients exposed to EVAR compared to open repair. This also translated into a substantial benefit in being a more cost-effective strategy with a >90% probability at all levels of willingness to pay for a quality-adjusted life year gain (47).

Lastly, a Cochrane database analysis did not show a clear benefit for either strategy (30-day mortality: EVAR vs. Open, OR 0.88, 95% CI 0.66–1.16) though complications were less likely when a stent-graft approach was used (any complication: Open vs. EVAR, OR 2.38, 95% CI 0.34–16.53) although the assessed studies were judged to be moderate-to-low quality evidence. Despite the mixed results, current societal guidelines support an ‘EVAR first’ strategy when feasible for rAAA management.

A unique but rare scenario that can be encountered with rAAA management is an associated aorto-enteric or aorto-caval fistula (AEF/ACF). Although the focus of this analysis was to chronicle contemporary emergency management of degenerative aneurysm disease, it is important to highlight that these presentations are possible when surgeons treat non-elective aneurysm patients. A high index of suspicion is necessary to address these two unique pathologies and contemporary CT angiography with delayed venous phasing is helpful to inform impressions about the underlying anatomic morphology of the aneurysm.

Management for AEF generally mandates some form of aneurysm extirpation (either single or staged operation with either in-situ or extra-anatomic vascular reconstruction) and some series have documented use of endoluminal techniques as bridging adjuncts to physiologically stabilize the acute GI bleeding problem (if there is sufficient landing zone relative to the renal arteries). However, endoluminal strategies are generally not used as a destination therapy in these cases. In contrast, EVAR can be used to definitively manage an ACF through primary exclusion of the aneurysm. If open repair occurs and an ACF is present, the repair of the aorta-inferior vena cava communication should occur from inside the aneurysm sac with sponge stick control.

## Conclusion

In conclusion, contemporary outcomes after the surgical management of rAAA are underscored by the longitudinal trends in patient selection, perioperative and postoperative resuscitation, as well as technological advancements that have resulted in improved results with an increasing focus on patient-centered care. Interestingly, more recent evidence has suggested that the incidence of rAAA is decreasing over time; however, this challenging clinical problem inevitably persists in current practice. As such, the contemporary vascular surgeon needs to remain up to date on the evolving evidence-base surrounding rAAA management. The current review provides a comprehensive perspective on the relevant aspects of emergency AAA management that spans the pre-hospital, intraoperative and postoperative domains of care.

Since the first descriptions of successful rAAA repair in the mid-20th century, profound changes have occurred that have largely benefited patients and improved outcomes. Although the debate surrounding the superiority of EVAR vs. Open repair remains unresolved, there are a number of other advancements that have been adopted globally including increasing efforts for regionalization, more precise care coordination, judicious physiological resuscitation, utilization of high-quality imaging, as well as process of care delivery paradigms. In aggregate, these transformative efforts now highlight the modern management of ruptured AAA. Future directions will need to focus on better delineating the rightful role of EVAR vs. Open repair in the emergent setting, while also leveraging new technologies including artificial intelligence to enhance processes of care, patient allocation, and selection to further improve rAAA outcomes. Regardless, these comprehensive aggregate advances in rAAA care document a success story in clinical aneurysm care delivery and highlight the collective efforts in contemporary healthcare to improve aortic surgery.
